# [^18^F]THK5317 imaging as a tool for predicting prospective cognitive decline in Alzheimer’s disease

**DOI:** 10.1038/s41380-020-0815-4

**Published:** 2020-07-03

**Authors:** Konstantinos Chiotis, Irina Savitcheva, Konstantinos Poulakis, Laure Saint-Aubert, Anders Wall, Gunnar Antoni, Agneta Nordberg

**Affiliations:** 1 Nordberg Translational Molecular Imaging Lab, Division of Clinical Geriatrics, Center for Alzheimer Research, Department of Neurobiology, Care Sciences and Society, Karolinska Institutet, Stockholm, Sweden; 2 Theme Neurology, Karolinska University Hospital, Stockholm, Sweden; 3 Medical Radiation Physics and Nuclear Medicine, Karolinska University Hospital, Stockholm, Sweden; 4 Westman neuroimaging group, Division of Clinical Geriatrics, Center for Alzheimer Research, Department of Neurobiology, Care Sciences and Society, Karolinska Institutet, Stockholm, Sweden; 5 Toulouse NeuroImaging Center, University of Toulouse, Inserm, UPS, Toulouse, France; 6 Section for Nuclear Medicine and PET, Department of Surgical Sciences, Uppsala University, Uppsala, Sweden; 7 Department of Medicinal Chemistry, Uppsala University, Uppsala, Sweden; 8 Theme Aging, Karolinska University Hospital, Stockholm, Sweden

**Keywords:** Psychiatric disorders, Predictive markers, Prognostic markers

## Abstract

Cross-sectional studies have indicated potential for positron emission tomography (PET) in imaging tau pathology in Alzheimer’s disease (AD); however, its prognostic utility remains unproven. In a longitudinal, multi-modal, prognostic study of cognitive decline, 20 patients with a clinical biomarker-based diagnosis in the AD spectrum (mild cognitive impairment or dementia and a positive amyloid-beta PET scan) were recruited from the Cognitive Clinic at Karolinska University Hospital. The participants underwent baseline neuropsychological assessment, PET imaging with [^18^F]THK5317, [^11^C]PIB and [^18^F]FDG, magnetic resonance imaging, and in a subgroup cerebrospinal fluid (CSF) sampling, with clinical follow-up after a median 48 months (interquartile range = 32:56). In total, 11 patients declined cognitively over time, while 9 remained cognitively stable. The accuracy of baseline [^18^F]THK5317 binding in temporal areas was excellent at predicting future cognitive decline (area under the receiver operating curve 0.84–1.00) and the biomarker levels were strongly associated with the rate of cognitive decline (β estimate −33.67 to −31.02, *p* < 0.05). The predictive accuracy of the other baseline biomarkers was poor (area under the receiver operating curve 0.58–0.77) and their levels were not associated with the rate of cognitive decline (β estimate −4.64 to 15.78, *p* > 0.05). Baseline [^18^F]THK5317 binding and CSF tau levels were more strongly associated with the MMSE score at follow-up than at baseline (*p* < 0.05). These findings support a temporal dissociation between tau deposition and cognitive impairment, and suggest that [^18^F]THK5317 predicts future cognitive decline better than other biomarkers. The use of imaging markers for tau pathology could prove useful for clinical prognostic assessment and screening before inclusion in relevant clinical trials.

## Introduction

Alzheimer’s disease (AD) is characterized by the presence of insoluble fibrillar deposits of amyloid-beta in plaque structures and tau protein in neurofibrillary tangles. The development of positron emission tomography (PET) tracers [1], and cerebrospinal fluid (CSF) and blood assays [2, 3], for measuring in vivo levels of amyloid-beta pathology reconceptualized the diagnosis of AD by allowing, for the first time, diagnosis in vivo in patients with objective cognitive impairment and abnormal levels of amyloid-beta, at both the mild cognitive impairment (MCI) stage and the dementia stage (now called MCI or dementia due to AD, respectively) [4–6]. Longitudinal studies have, however, stressed that although the amyloid-beta biomarkers are highly sensitive to the presence of amyloid-beta pathology, they have limited specificity in determining which patients with MCI will decline cognitively over time and develop dementia of the Alzheimer’s type [7]. This finding, together with the high frequency of amyloid-beta-positive, cognitively normal elderly [8, 9], questions the deterministic role of a positive amyloid-beta biomarker result in predicting AD-related cognitive decline in a clinical setting.

Studies have indicated potential for tau PET in differentiating between cognitively normal elderly and patients with AD [10, 11], for its strong relationship with cognitive impairment [12–17], and in discriminating between different subtypes of AD [18]. However, the ability of tau PET to predict which patients will decline cognitively over time remains to be determined. In this study, we followed patients with AD who had undergone baseline imaging with the first generation tau PET tracer [^18^F]THK5317 [10, 19, 20]. We wanted to determine the accuracy of predicting future cognitive decline from the extent of baseline tracer binding, and to compare this with the predictive accuracy using baseline [^18^F]FDG and [^11^C]PIB PET, and clinical CSF, neuropsychological and structural atrophy markers.

## Materials and methods

### Study participants

Twenty patients with a clinical diagnosis of MCI or dementia due to AD who had previously participated in baseline multi-modal investigations were included in this longitudinal prognostic study. All patients had been referred to the Cognitive Clinic at Theme Aging, Karolinska University Hospital, Stockholm, Sweden, for memory assessment. The procedures for clinical assessment and patient recruitment are detailed elsewhere [10]. At baseline, nine patients were diagnosed with dementia due to AD (that is, probable AD and a positive [^11^C]PIB PET scan) and 11 with MCI due to AD (that is, amnestic multi-domain MCI and a positive [^11^C]PIB PET scan) [4, 6]. All patients were followed up clinically at regular intervals to determine the extent of clinically evident cognitive decline over time (as described in the Neuropsychological assessment section). For the purposes of this study we focused on the most recent follow-up assessments (those that were available at 1st of November 2019). The median follow-up for our patient group was 48.02 months (interquartile range = 32.04:56.33).

The study was approved by the Regional Human Ethics committee in Stockholm, and the Radiation Safety committee of Uppsala University Hospital, Sweden. All participants and their caregivers provided written informed consent prior to the investigation and all procedures were in accordance with the ethical standards of the Institutional and National Research Committee and with the 1964 Helsinki Declaration and its later amendments, or comparable ethical standards.

### Neuropsychological assessment

All patients underwent neuropsychological assessment at baseline. This included assessment of global cognition using the Mini-Mental State Examination (MMSE), and of episodic memory using, among others [12], the Rey Auditory-Verbal Learning (RAVL) encoding subtest, expressed as *z*-scores, in comparison with results from a reference group of healthy controls [21].

The patients were assessed again using MMSE at follow-up, with the exception of one patient with a diagnosis of AD dementia at baseline who could not complete the assessment because of severe cognitive decline over the follow-up interval. Cognitive decline over time was defined as a decrease in MMSE score of ≥1.5 MMSE units per year [15, 22, 23], coupled with evidence of cognitive deterioration in multiple domains over the follow-up interval based on the medical history from patients and caregivers. The participants were subsequently divided into two subgroups (those who were cognitively stable vs those with cognitive decline). The patient who could not complete follow-up MMSE assessment because of severe cognitive impairment was placed in the cognitive decline group. This patient, who became eligible for nursing-home care during the follow-up interval as a result of requiring extensive assistance with the activities of daily living, was not included in analyses employing MMSE measures as the dependent variable.

### Image analysis

At baseline, all patients had undergone [^18^F]THK5317, [^11^C]PIB and [^18^F]FDG PET imaging, and a T1-MRI sequence, as detailed elsewhere [10]. Individual dynamic baseline [^18^F]THK5317 PET (0-60 min) images were co-registered onto the individual T1-MRI image and kinetic modelling using the reference Logan graphical method was applied to create parametric distribution volume ratio (DVR) images, using the cerebellar grey matter as reference (PMOD v.3.5). In region-based analyses, for minimizing the spill-over effect on the signal because of atrophy, MRI-based partial volume correction was applied to the dynamic [^18^F]THK5317 PET images, based on individual T1 scans, prior to kinetic modelling using the Muller-Gartner method (PMOD v.3.5) [12]. For voxel-based analyses of the [^18^F]THK5317 data, non-partial volume corrected data was used, since the application of correction resulted to the amplification of the noise in extra-cerebral areas. Summation [^11^C]PIB (40-60 min) and [^18^F]FDG (30–45 min) PET images were co-registered onto the individual T1-MRI images (SPM8). Standard uptake value ratio (SUVR) images were created using the cerebellar grey matter as the reference for [^11^C]PIB and the pons for [^18^F]FDG.

For regional quantification, we used regions of interest (ROIs) derived from the Harvard-Oxford probabilistic atlas (FSL), spatially warped in each patient’s native T1-MRI space, after application of an individual grey matter mask, as previously described [12]. Based on the atlas, bilateral composite ROIs were created for the quantification of the [^18^F]THK5317 and [^18^F]FDG parametric images: inferior temporal gyrus, middle temporal gyrus, superior temporal gyrus, lateral parietal lobe, occipital lobe, frontal lobe and precuneus. Binding was not assessed in ROIs with high MAO-B loads (e.g., striatum, thalamus, medial temporal lobe, cingulate gyrus), given the affinity of [^18^F]THK5317 for the MAO-B enzyme [24]. A composite neocortical ROI was created for quantifying [^11^C]PIB binding.

An experienced neuroradiologist rated the T1-MRI sequence for assessing the medial temporal lobe atrophy (MTA) score [25]. The left and right MTA scores were averaged.

### CSF measurements

CSF samples were obtained under non-fasting conditions via lumbar puncture from 17 participants at baseline. Levels of Aβ_1-42_, and p-tau_181p_ were determined in 16 participants, and t-tau in all 17 participants using commercially available sandwich ELISAs (Innogenetics, Ghent, Belgium).

### Statistical analyses

Wilcoxon rank sum and chi-squared tests were used for comparing the clinical characteristics of patients who remained cognitively stable with those of patients who declined cognitively over the follow-up interval, (uncorrected *p* < 0.05).

The areas under the receiver operating characteristic curves (AUC) were calculated to assess the accuracy of the baseline biomarker levels [e.g., regional tracer binding/uptake (DVR or SUVR), neuropsychological and CSF measurements, and atrophy ratings] in differentiating patients who remained cognitively stable from those who declined cognitively over time. The Youden index was used for determining optimal cut-off points at a regional level.

Linear models were used for evaluating the association between baseline biomarker levels and the decrease in MMSE score over time (ΔMMSE; follow-up minus baseline), after adjusting for relevant covariates (Eq. (1)). Linear mixed-effects models were used for assessing the effect of the interaction between the baseline biomarker levels and time from baseline on the MMSE scores (baseline or follow-up), after adjusting for relevant covariates and accounting for repeated measurements (Eq. (2)). The follow-up assessments were assigned a time from baseline corresponding to the difference in months between baseline (time from baseline = 0) and the follow-up investigations. All models were also replicated after the addition of baseline diagnosis (MCI due to AD or dementia due to AD) as covariate, and the results are presented in Supplementary Figs. 1, 2. No collinearity was detected in the models.

Bonferroni-corrected alpha levels for all the models assessing the effects of regional tracer binding/uptake ([^18^F]THK5317 and [^18^F]FDG) were based on the number of assessed ROIs (*n* = 7; Bonferroni-corrected *p* < 0.05). The models assessing the effects of clinical biomarkers (cognitive, CSF and atrophy markers, and composite [^11^C]PIB binding) were not corrected for multiple comparisons (uncorrected *p* < 0.05).

All regional analyses for [^18^F]THK5317 binding were replicated in the subgroup of patients with available CSF measures (*n* = 16) for comparison (Supplementary Fig. 3).

All the above-mentioned statistical analyses were carried out using R 3.6.0 software.

1 $$	\Delta{\rm{MMSE}}\;\left( {\rm{follow}} {\hbox{-}} {\rm{up}}\;{\rm{minus}}\;{\rm{baseline}} \right)\\ 	\,= {\rm{Baseline}}\; {\rm{biomarker}}\; {\rm{levels}} \\ 	\quad\, +{\rm{Time}}\; {\rm{interval}}\; {\rm{between}}\; {\rm{MMSE}}\; {\rm{investigations}} + {\rm{Age}},$$

2 $$	{\rm{MMSE}}\; \left( {{\rm{baseline}}\;{\rm{or}}\;{\rm{follow}} {\hbox{-}} {\rm{up}}} \right)\\ 	\,= {\rm{Baseline}}\;{\rm{biomarker}}\;{\rm{levels}} \\ 	\, \quad + {\rm{Time}}\;{\rm{from}}\;{\rm{baseline}} + {\rm{Age}} + {\rm{Baseline}}\;{\rm{biomarker}}\;{\rm{level}}\; \\ 	\, \quad \sim \;{\rm{Time}}\;{\rm{from}}\;{\rm{baseline}}\;\left( {{\rm{interaction}}} \right)\\ 	\quad \,+ {\rm{Random}}\;{\rm{intercept}}\;\left( {{\rm{participant ID}}} \right).$$

### Statistical analyses—voxel-based comparisons

Following the above-mentioned pre-processing steps, we performed spatial normalization of all voxel-based maps for [^18^F]THK5317, [^18^F]FDG and [^11^C]PIB into the MNI space using the individual transformation matrices from the T1-MRI segmentation step (SPM8). A Gaussian smoothing kernel (FWHM = 8 mm in all directions) was applied to the images. An explicit grey matter mask was used to restrict the voxel-based analyses to GM regions.

The AUC were calculated at a voxel level to assess the accuracy of the baseline tracers’ binding (DVR or SUVR) in differentiating patients who remained cognitively stable from those who declined cognitively over time using VoxelStats 1.1 [26].

A multiple regression design was used for implementing correlation analyses at a voxel level. Briefly, the association between baseline tracer binding/uptake (DVR or SUVR) and the decrease in MMSE score (ΔMMSE) over time was evaluated after adjusting for the time interval between MMSE investigations (continuous variable) and age at baseline (continuous variable). The models were also evaluated after the addition of baseline diagnosis as covariate (dummy variable), and the results are presented in Supplementary Fig. 1. The relevant contrasts for ΔMMSE were evaluated (positive correlations for [^18^F]FDG SUVR, and negative for [^18^F]THK5317 DVR and [^11^C]PIB SUVR).

Separate multiple regression designs were used for evaluating the relationship between baseline tracers’ binding/uptake (DVR or SUVR) and (1) MMSE at baseline and (2) MMSE at follow-up, in two separate models. The relevant contrasts for MMSE were evaluated (positive for [^18^F]FDG SUVR, and negative for [^18^F]THK5317 DVR and [^11^C]PIB SUVR).

All the above-mentioned voxel-based statistical analyses involving regression models were carried out using SPM8 software. For significance testing, no correction for multiple comparisons was applied at the voxel level (*p* < 0.001). A correction for multiple comparisons was applied at a cluster level using the family-wise error rate (FWE-cluster-corrected, *p* < 0.05). The results of the voxel-based comparisons were projected onto group average cortical surfaces using BrainNet Viewer 1.61 software [27].

## Results

### Study participants

At follow-up, seven patients with a diagnosis of MCI due to AD and two with dementia due to AD remained clinically cognitively stable (cognitively stable group, *n* = 9; ΔMMSE <1.5 units/year). Four patients with a baseline diagnosis of MCI due to AD and seven patients with a baseline diagnosis of dementia due to AD experienced further cognitive decline over time (cognitive decline group, *n* = 11; ΔMMSE ≥1.5 units/year). In total, 4 of 11 patients in the cognitive decline group were admitted to nursing home over the follow-up interval.

The follow-up interval was more than 24 months (median interval = 48.02 months; interquartile range = 32.04:56.33) for all except one patient, who had a baseline diagnosis of AD dementia and was in the cognitively stable group (ΔMMSE = 1). This patient’s latest cognitive assessment was 17 months after baseline and the patient died shortly after from unrelated causes. The clinical characteristics of the patients who remained cognitively stable vs those who experienced cognitive decline over time are summarized in Table 1.

**Table 1:** Demographic and clinical characteristics of the patient sample.

	All	Cognitively stable	Cognitive decline	p (unc).
**Clinical data**				
*N*	20	9	11	–
Age (years)	68.5 [63.0: 74.0]	65.0 [65.0:74.0]	70 [59.5:74.0]	0.674
Gender (m/f), n	7/13	5/6	2/7	0.279
Education (years)	12.5 [10.75:14.25]	12 [10:12]	14 [12.5:15.5]	0.116
ApoE ε4 (carriers/non-carriers), n	14/5^a^	5/4	9/1^a^	0.089
**Clinical diagnosis at baseline**				
(MCI due to AD / dementia due to AD), n	11/9	7/2	4/7	0.064
**Clinical biomarker data**				
Composite [^11^C]PIB (SUVR)	1.75 [1.67:1.95]	1.68 [1.65:1.87]	1.78 [1.72:2.03]	0.175
CSF Aβ_1-42_ (pg/ml)	516.00^b^ [470.75:583.25]	608.50^a^ [475.00:851.00]	491.00^c^ [470.75:523.25]	0.195
CSF Aβ_1-42_/t-tau ratio	1.22^b^ [0.82:1.80]	1.60^a^ [1.16:1.95]	0.94^c^ [0.70:1.31]	0.130
CSF t-tau (pg/ml)	465.00^c^ [315.00:639.00]	465.00 [301.00:492.00]	546.00^c^ [368.00:667.75]	0.370
CSF p-tau_181p_ (pg/ml)	75.00^b^ [50.75:99.25]	62.50^a^ [47.75:89.00]	82.00^c^ [57.00:99.25]	0.382
MTA	1.5 [1.0:2.0]	1.0 [1.0:1.5]	1.5 [1.00:2.25]	0.240
**Neuropsychological data at baseline**				
MMSE (baseline)	25.5 [23.0:29.0]	29.0 [26.0:30.0]	25.0 [23.0:26.0]	0.055
RAVL encoding (baseline), *z*-score	−2.19 [−2.43: −0.99]	−1.76 [−2.24: −0.51]	−2.34 [−2.48: −1.81]	0.128
**Follow-up assessment**				
MMSE (follow-up)	21.0^d^ [14.0:25.5]	26.0 [24.0:27.0]	14.0^d^ [6.5:15.0]	<0.001
ΔMMSE	6.0 [2.0:11.5]^d^	2.0 [1.0:2.0]	11.5^d^ [9.3:16.5]	<0.001
Follow-up interval (months)	48.02 [32.04:56.33]	52.97 [28.77:56.43]	46.47 [33.15:55.12]	0.882

The data are presented as median [interquartile range], unless otherwise indicated.

*ApoE* apolipoprotein E, *f* female, *m* male, *unc.* uncorrected.

^a^Data missing for one patient.

^b^Data missing for four patients.

^c^Data missing for three patients.

^d^Data missing for one patient who could not complete the follow-up MMSE assessments because of severe cognitive decline.

### Accuracy of the biomarker levels for predicting cognitive decline over time

**Fig. 1:**
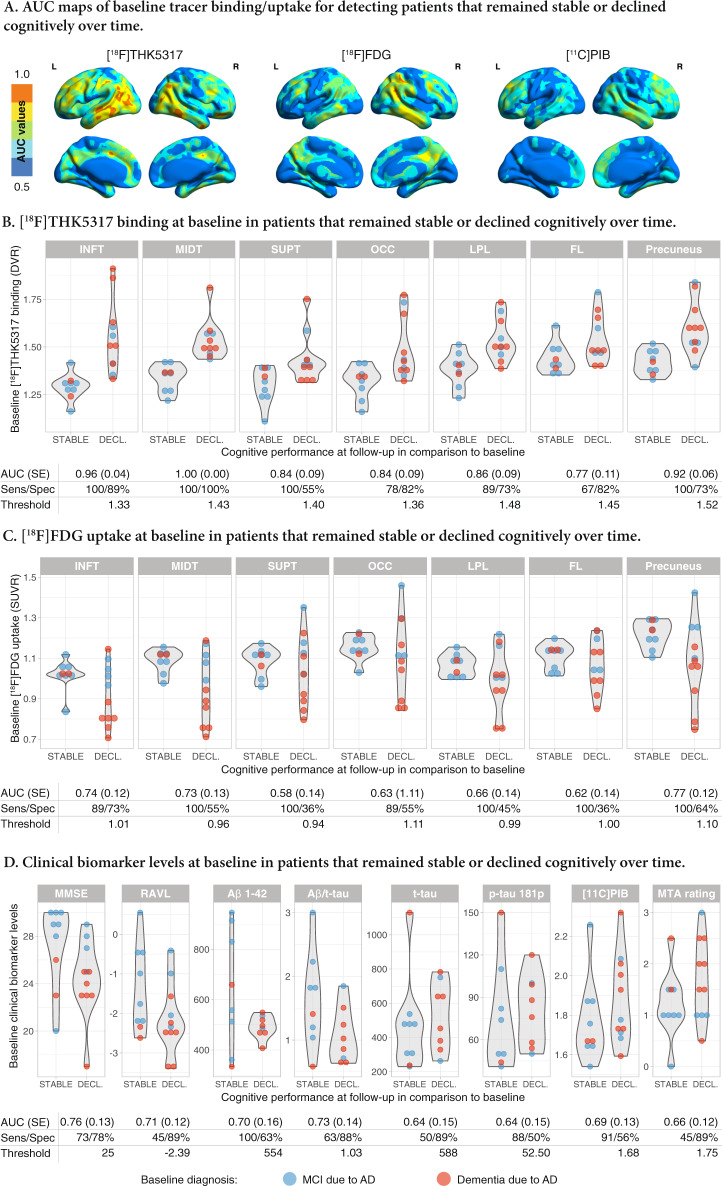
Accuracy of baseline biomarker levels in detecting patients whose cognitive performance declined over time (cognitively stable vs cognitive decline groups). **a** Voxel-based AUC maps of the binding/uptake of the tracers. Dot and violin plots showing the levels of (**b**) regional baseline [^18^F]THK5317 binding, (**c**) regional baseline [^18^F]FDG uptake, and (**d**) clinical baseline biomarker levels in patients who remained cognitively stable (STABLE) and those who declined cognitively (DECL) over time. The calculated AUC and the sensitivity and specificity of the optimal cut-off points for classifying patients who remained cognitively stable or declined cognitively over time are shown below the respective dot and violin plots. SE standard error, FL frontal lobe, INFT inferior temporal gyrus, LPL lateral parietal lobe, MIDT middle temporal gyrus, OCC occipital lobe, SUPT superior temporal gyrus.

**Fig. 2:**
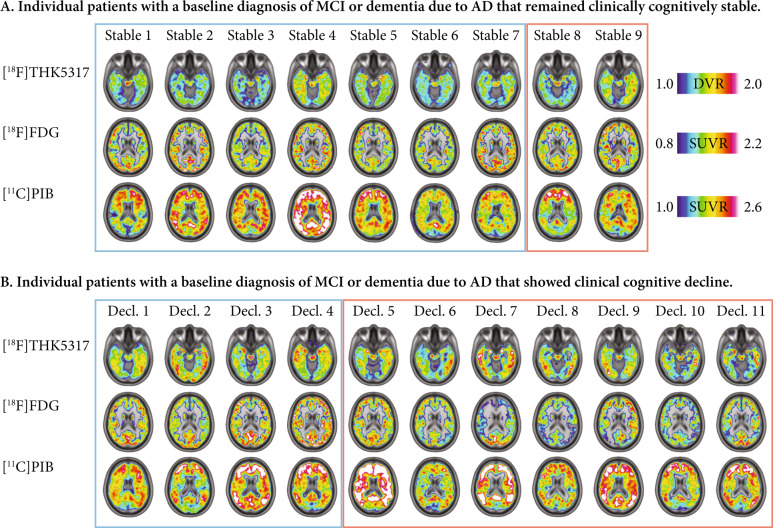
Baseline PET scans of the patient sample. Baseline [^18^F]THK5317 (DVR), [^18^F]FDG (SUVR) and [^11^C]PIB (SUVR) PET scans from the individual patients with baseline diagnoses of MCI or dementia due to AD (blue and red outlined boxes, respectively), who (**a**) remained cognitively stable vs those who (**b**) experienced cognitive decline over time (Decl).

At a voxel level, the accuracy of baseline [^18^F]THK5317 binding levels in bilateral temporoparietal areas for predicting subsequent cognitive decline in the patient sample (cognitively stable vs cognitive decline groups) was excellent (*n* = 20, AUC > 90%), while the accuracy of baseline [^18^F]FDG uptake was much more moderate and accurate predictions were only seen in unilateral temporoparietal areas (*n* = 20) (Fig. 1a). Baseline [^11^C]PIB binding levels showed poor predictive accuracy (*n* = 20). The [^18^F]THK5317 DVR, [^18^F]FDG SUVR and [^11^C]PIB SUVR images from the patients with baseline diagnoses of MCI or dementia due to AD who remained cognitively stable vs those who experienced cognitive decline over time are shown in Fig. 2.

At a regional level, baseline [^18^F]THK5317 binding in the middle temporal gyrus was 100% accurate in predicting cognitive decline (*n* = 20, AUC: 1.00; sensitivity 100%; specificity 100%) while binding in the inferior temporal gyrus was marginally less accurate, followed by the precuneus (Fig. 1b). The predictive accuracy of baseline [^18^F]FDG uptake was highest in the precuneus (*n* = 20, AUC: 0.77; sensitivity 100%; specificity 64%; Fig. 1c). The predictions from all the clinical biomarkers at baseline were less accurate (*n* = 20 for MMSE, RAVL encoding, composite [^11^C]PIB binding and MTA rating; *n* = 16 for CSF Aβ_1-42_ and p-tau_181p_; *n* = 17 for CSF t-tau; Fig. 1d).

### Association of baseline biomarker levels with future decrease in MMSE score

**Fig. 3:**
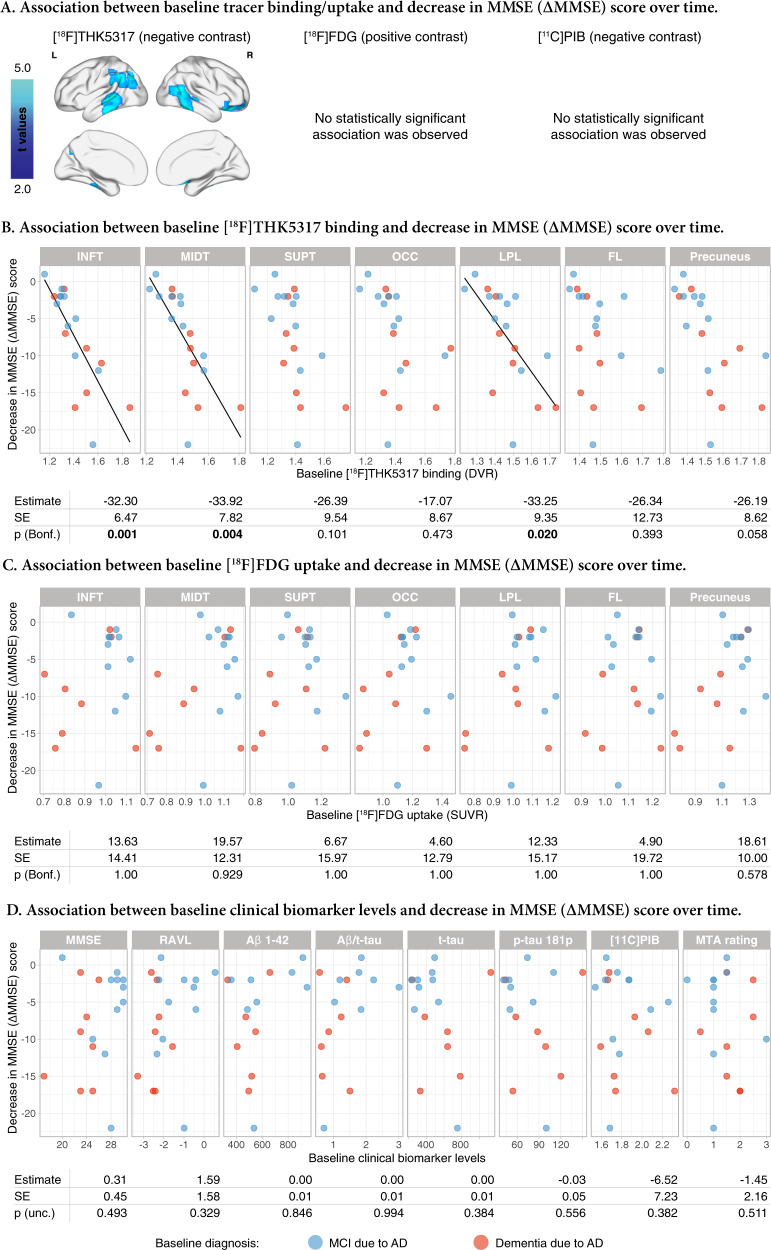
Association between baseline biomarker levels and decreased MMSE score (ΔMMSE) over time, after adjusting for age and interval between MMSE assessments. **a** Voxel-based multiple regression of the relationship between the baseline binding/uptake of the tracers and the decrease in MMSE score. Scatterplots showing the relationships between (**b**) regional baseline [^18^F]THK5317 binding, (**c**) regional baseline [^18^F]FDG uptake, and (**d**) clinical baseline biomarker levels and decreased MMSE scores. The estimated effect, the standard error (SE) and the linear model p values are shown below the respective scatter plots. Bonferroni-corrected (Bonf.) p values based on the number of regions investigated (*n* = 7) are presented for regional tracer binding/uptake results. Uncorrected (unc.) values are presented for the clinical biomarker results. FL frontal lobe, INFT inferior temporal gyrus, LPL lateral parietal lobe, MIDT middle temporal gyrus, OCC occipital lobe, SUPT superior temporal gyrus.

At a voxel level, baseline [^18^F]THK5317 binding in temporal areas was significantly associated negatively with cognitive decline (*n* = 19, FWE-cluster-corrected *p* < 0.05), after adjusting for relevant covariates (Fig. 3a). No statistically significant association was found between baseline levels of [^18^F]FDG uptake or [^11^C]PIB binding and cognitive decline (*n* = 19, FWE-cluster-corrected *p* > 0.05).

At a regional level, baseline [^18^F]THK5317 binding in temporoparietal areas was associated with a significant decrease in MMSE score, after adjusting for relevant covariates (*n* = 19, Bonferroni-corrected *p* < 0.05; Fig. 3b). No statistically significant association was found between baseline regional levels of [^18^F]FDG uptake and a decrease in MMSE score (*n* = 19, Bonferroni-corrected *p* > 0.05; Fig. 3c). Nor was a statistically significant association found between the baseline levels of the clinical biomarkers and a decrease in MMSE score (*n* = 19 for MMSE, RAVL encoding, composite [^11^C]PIB binding and MTA rating; *n* = 15 for CSF Aβ_1-42_ and p-tau_181p_; *n* = 16 for CSF t-tau; uncorrected *p* > 0.05 for all; Fig. 3d).

### Association of baseline biomarker levels with MMSE score longitudinally

**Fig. 4:**
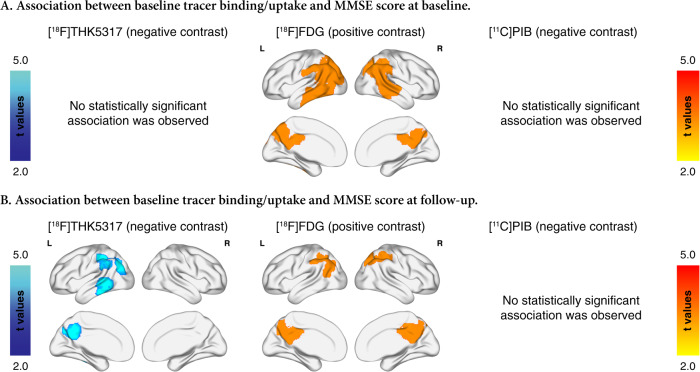
Association between baseline tracer binding/uptake and MMSE at the two time points. Voxel-based multiple regression of the relationship between the baseline binding/uptake of the tracers and the MMSE score at (**a**) baseline, and (**b**) follow-up.

At a voxel level, baseline [^18^F]THK5317 binding in temporoparietal areas was associated negatively with follow-up but not with baseline MMSE scores (*n* = 19, FWE-cluster-corrected *p* < 0.05) (Fig. 4a, b). Baseline [^18^F]FDG uptake in temporoparietal areas was significantly associated with baseline MMSE scores, and in more restricted parietal areas with follow-up MMSE scores (*n* = 19, FWE-cluster-corrected *p* < 0.05). Baseline [^11^C]PIB binding was not associated with baseline or follow-up MMSE scores (*n* = 19, FWE-cluster-corrected *p* < 0.05).

**Fig. 5:**
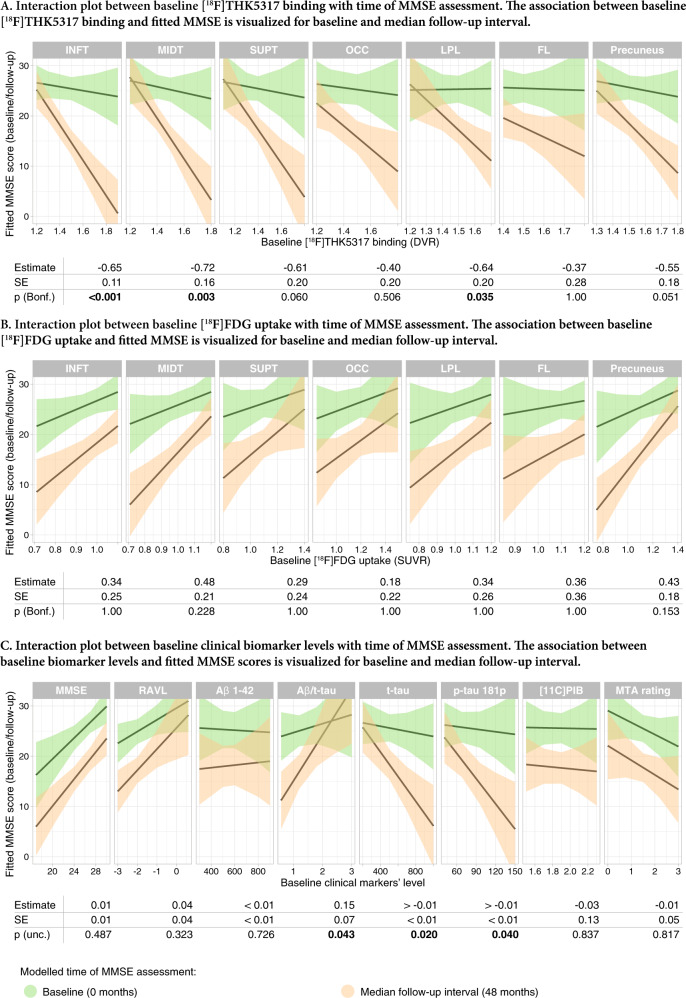
Interaction plots of the relationship between baseline biomarker levels and MMSE scores at baseline and follow-up (biomarker level x time) as fitted by linear-mixed effects models after adjusting for age. For simplifying the interpretation of this two-way interaction, the time point was treated as the moderator of the relationship between baseline biomarker levels and MMSE. Discreet values for the follow-up time points were selected from the output of the model for visualization based on the most frequent follow-up intervals among the participants (baseline 0 months, and median follow-up interval of 48 months). The visualized modelled-fitted values were based on the whole patient sample. Differential relationships between baseline (**a**) regional [^18^F]THK5317 binding, (**b**) regional [^18^F]FDG binding, and (**c**) clinical biomarker levels and MMSE score at the different time points. The estimated interaction effect, the standard error (SE) and the *p* values are shown below the respective interaction plots. Bonferroni-corrected (Bonf.) *p* values based on the number of regions investigated (*n* = 7) are presented for regional tracer binding/uptake results. Uncorrected (unc.) values are presented for the clinical biomarker results. FL frontal lobe, *INFT* inferior temporal gyrus, LPL lateral parietal lobe, MIDT middle temporal gyrus, OCC occipital lobe, SUPT superior temporal gyrus.

In order to assess whether the relationship of biomarker levels and MMSE score is moderated by the time point of MMSE evaluation, we assessed the interaction term [biomarker levels x time point] for the different biomarker modalities, after adjusting for relevant covariates, as described above. The interaction term was significant for baseline regional [^18^F]THK5317 binding in temporoparietal ROIs (*n* = 19, Bonferroni-corrected *p* < 0.05) and for baseline CSF levels of Aβ_1-42_/t-tau ratio, t-tau, and p-tau_181p_ (*n* = 16 for CSF t-tau and *n* = 15 for CSF p-tau_181p_; uncorrected *p* < 0.05). More specifically, the interaction plots (Fig. 5a–c) indicated that baseline [^18^F]THK5317 binding in temporoparietal areas was more strongly negatively associated with MMSE scores at follow-up than at baseline. A similar effect was detected for CSF measures of tau; however, the modelled estimates of the effect were small. The interaction term was not significant for the [^18^F]FDG uptake and the other clinical biomarker levels.

## Discussion

Several reports from in vivo and post-mortem data have highlighted the close relationship between cross-sectional measures of tau pathology and cognition in AD [9, 12, 15–17, 28, 29]. Our data highlight that baseline [^18^F]THK5317 PET showed higher prognostic accuracy to predict a future decline in cognitive performance, relative to other clinically available biomarkers. Furthermore, our results suggest the existence of a temporal dissociation between tau pathology and cognitive decline, with [^18^F]THK5317 binding relating stronger with prospective rather than cross-sectional cognitive performance.

In our study, baseline [^18^F]THK5317 binding predicted the subsequent stability of or decline in cognitive performance over time in patients with a clinical diagnosis in the AD spectrum (MCI or dementia due to AD) with excellent accuracy. Furthermore, there was a strong association between the extent of tracer binding at baseline and the extent of cognitive decline (ΔMMSE) over time, indicating that the extent of baseline tracer binding could give an indication of the rate of cognitive decline in patients with objective cognitive impairment and a positive amyloid-beta biomarker. It is therefore likely that the presence of tau pathology in neocortical association areas will have a negative impact on cognition over time. These results support the currently accepted hypothesis that tau pathology is a pathophysiological marker of AD and not simply a downstream marker that only correlates with cross-sectional measures of the stage of the disease [5]. Moreover, our work underlines the utility of tau PET imaging for early identification of patients at risk of cognitive decline in the AD spectrum, which would enable accurate prognosis in a clinical setting and inclusion of appropriate participants in anti-AD clinical trials.

In contrast, in our sample as in previous reports [30, 31], the accuracy of baseline [^18^F]FDG levels in predicting which patients would decline cognitively over time was only fair, and there was no relationship between the extent of tracer uptake and the rate of subsequent cognitive decline. These results, together with the current evidence for a strong relationship between [^18^F]FDG and global cognitive measures cross-sectionally in AD [12, 32], encourage its use mainly as a staging marker for the disease [5]. Figs. 1b and c and 2 demonstrate that [^18^F]FDG uptake levels, but not [^18^F]THK5317 binding levels, are better for discriminating between the stages of AD (MCI vs dementia due to AD) than for predicting cognitive decline. As expected from previous studies, the baseline clinical cognition markers, and the gross clinical atrophy markers, which are probably the most downstream markers of AD [33], were less accurate in predicting cognitive decline than the [^18^F]FDG levels [32].

Comparison of the predictive accuracy of [^11^C]PIB or CSF Aβ_1-42_ with that of [^18^F]THK5317 was complex in this study, given the known amyloid-beta positivity of all participants. From our analyses, however, it is evident that in the presence of a positive amyloid PET scan, further increases in the levels of amyloid tracer binding will not be predictive of future cognitive decline, at least at clinically relevant follow-up intervals. Interestingly, and in agreement with previous studies, only 11 of 20 (55%) patients with amyloid-positive PET scans experienced cognitive decline, and these were mainly those in the later stages of cognitive impairment (4 of 11 patients with MCI declined cognitively vs 7 of 9 patients with dementia) [34]. This observation adds to the cumulative evidence questioning the deterministic role of a positive amyloid-beta biomarker in patients with cognitive impairment [35], and highlights the need for other more specific pathophysiological markers, such as [^18^F]THK5317, for determining which individuals are likely to undergo AD-related cognitive decline. This study, however, was not designed to compare the predictive accuracy of amyloid-beta with that of tau biomarkers, nor to assess their combination in a clinical setting.

In our additional analyses, we noted that [^18^F]THK5317 binding and, to a lesser extent, tau markers in the CSF (lower estimates, but in a more restricted sample size) were better associated with the MMSE score at follow-up than with the baseline MMSE values. These results contradict partly previous reports of strong correlations between cross-sectional measures of tau pathology and cognitive performance [18], and highlight that the relationship of tau pathology with prospective cognitive performance is stronger, which supports the prognostic utility of tau PET imaging. Furthermore, these results underline the presence of a temporal dissociation between the development of tau deposition and cognitive deficits—tau pathology appears to precede the development of cognitive impairment, as has been previously suggested in hypothetical models of the AD cascade [33]. In contrast, [^18^F]FDG uptake showed more extensive correlations with cross-sectional rather than prospective cognitive performance, which further stresses the staging role of this biomarker.

The most important limitation of this work lies in the fact that [^18^F]THK5317 interacts with the enzyme monoamine oxidase B (MAO-B), and the molecular basis behind this interaction has been proposed [19, 24, 36]. Similar non-intended binding to MAO-B has been described for the other first generation tau PET tracers, including [^18^F]Flortaucipir [24, 37, 38], and it is somewhat difficult, to date, to estimate the exact contributions of the different binding targets (e.g., tau deposits and MAO-B) in the in vivo tracer PET signal. However, earlier in vivo PET studies have shown that the regional pattern of MAO-B tracer (i.e., [^3^H]deuterium-L-deprenyl) binding is different from that of [^18^F]THK5317 binding, and MAO-B tracer binding appears to be less extensive in later stages of the AD (i.e., dementia-stage) in contrast to what seen with [^18^F]THK5317 [10, 39, 40]. This suggests that the contribution of the MAO-B component in [^18^F]THK5317 binding will be small in the neocortical areas that were evaluated, which is in agreement with the low load of MAO-B in these areas at autopsy [24, 41]. The small sample number of patients in the current sample and the inclusion of only a gross neuropsychological measure at follow-up (i.e., MMSE) stresses the need for validating the results in larger patient groups with the use of different neuropsychological measures. Since the CSF biomarker assessment at baseline was limited to a sub-sample of the patient group, it was not possible to compare accurately the performance of CSF biomarkers to that of other biomarker measurements, which were available for all patients. Being limited to the current patient group (discovery sample), the sensitivity/specificity measures for [^18^F]THK5317 binding should be interpreted with caution before the assessment of the biomarker’s accuracy in a validation cohort. Using cortical volume or thickness measures would be required for comparing fairly atrophy measures and [^18^F]THK5317 binding, although the use of different field strengths for MRI acquisitions in our patient sample precluded the use of such analyses for atrophy in this study. Although the acquisition of the [^18^F]FDG data was based on the European Association of Nuclear Medicine Neuroimaging Committee’s guidelines [42], the use of a relatively early, static protocol for [^18^F]FDG, in contrast to the dynamic acquisition of the [^18^F]THK5317 data, could act as a limitation in the comparison of prognostic accuracy between imaging modalities.

The strength of this study lies in the longitudinal evaluation of a clinically well characterized sample of patients with a diagnosis of AD (MCI or dementia due to AD). Furthermore, the patient group was followed for a clinically relevant time: more than two years. The accuracy of baseline levels of [^18^F]THK5317 in predicting prospective cognitive decline in these patients was excellent, in comparison to the fair to poor accuracy of baseline amyloid-beta deposition levels, baseline neurodegeneration, baseline CSF measures and baseline neuropsychological assessment. Our results emphasize that the use of tau PET could improve the outcomes of both clinical routines and ongoing trials by allowing the early, accurate identification of patients with AD who will experience cognitive decline.

## Supplementary information

Supplemental Material
