# Supplementary material for: [18F]THK5317 imaging as a tool for predicting prospective cognitive decline in Alzheimer’s disease
Source: Mol Psychiatry. 2020 Jul 3;26(10):5875–87. doi: 10.1038/s41380-020-0815-4 (PMC8758479; doi:10.1038/s41380-020-0815-4)

**Title**: [^18^F]THK5317 imaging as a tool for predicting prospective cognitive decline in Alzheimer’s disease

**Authors**: Konstantinos Chiotis MD, PhD ^1, 2^, Irina Savitcheva MD, PhD ^3^, Konstantinos Poulakis Msc ^4^, Laure Saint-Aubert, PhD ^1, 5^, Anders Wall PhD ^6^, Gunnar Antoni PhD ^7^, Agneta Nordberg MD, PhD ^1, 8^

**Affiliations**:

^1^ Nordberg Translational Molecular Imaging Lab, Division of Clinical Geriatrics, Center for Alzheimer Research, Department of Neurobiology, Care Sciences and Society, Karolinska Institutet, Stockholm, Sweden; ^2^ Theme Neurology, Karolinska University Hospital, Stockholm, Sweden; ^3^ Medical Radiation Physics and Nuclear Medicine, Karolinska University Hospital, Stockholm, Sweden; ^4^ Westman neuroimaging group, Division of Clinical Geriatrics, Center for Alzheimer Research, Department of Neurobiology, Care Sciences and Society, Karolinska Institutet, Stockholm, Sweden; ^5^ Toulouse NeuroImaging Center, University of Toulouse, Inserm, UPS, Toulouse, France; ^6^ Department of Surgical Sciences, Uppsala University, Uppsala, Sweden; ^7^ Department of Medicinal Chemistry, Uppsala University, Uppsala, Sweden; ^8^ Theme Aging, Karolinska University Hospital, Stockholm, Sweden.

**Corresponding author**:

Agneta Nordberg MD, PhD, professor

Karolinska Institutet, Dept NVS

Division of Clinical Geriatrics

Center for Alzheimer Research, Neo 7th floor

141 83 Huddinge, Sweden

Tel.: +46 8 52483532; E-mail: agneta.k.nordberg@ki.se

**Supplementary figures**

Supplementary Fig 1. Association between baseline biomarker levels and decreased MMSE score (ΔMMSE) over time, after adjusting for the baseline diagnosis, age and interval between MMSE assessments. (A) Voxel-based multiple regression of the relationship between the baseline binding/uptake of the tracers and the decrease in MMSE score. Scatterplots showing the relationships between (B) regional baseline [^18^F]THK5317 binding, (C) regional baseline [^18^F]FDG uptake, and (D) clinical baseline biomarker levels and decreased MMSE scores. The estimated effect, the standard error (SE) and the linear model p values are shown below the respective scatter plots. Bonferroni-corrected (Bonf.) p values based on the number of regions investigated (n=7) are presented for regional tracer binding/uptake results. Uncorrected (unc.) values are presented for the clinical biomarker results. FL, frontal lobe; INFT, inferior temporal gyrus; LPL, lateral parietal lobe; MIDT, middle temporal gyrus; OCC, occipital lobe; SUPT, superior temporal gyrus.


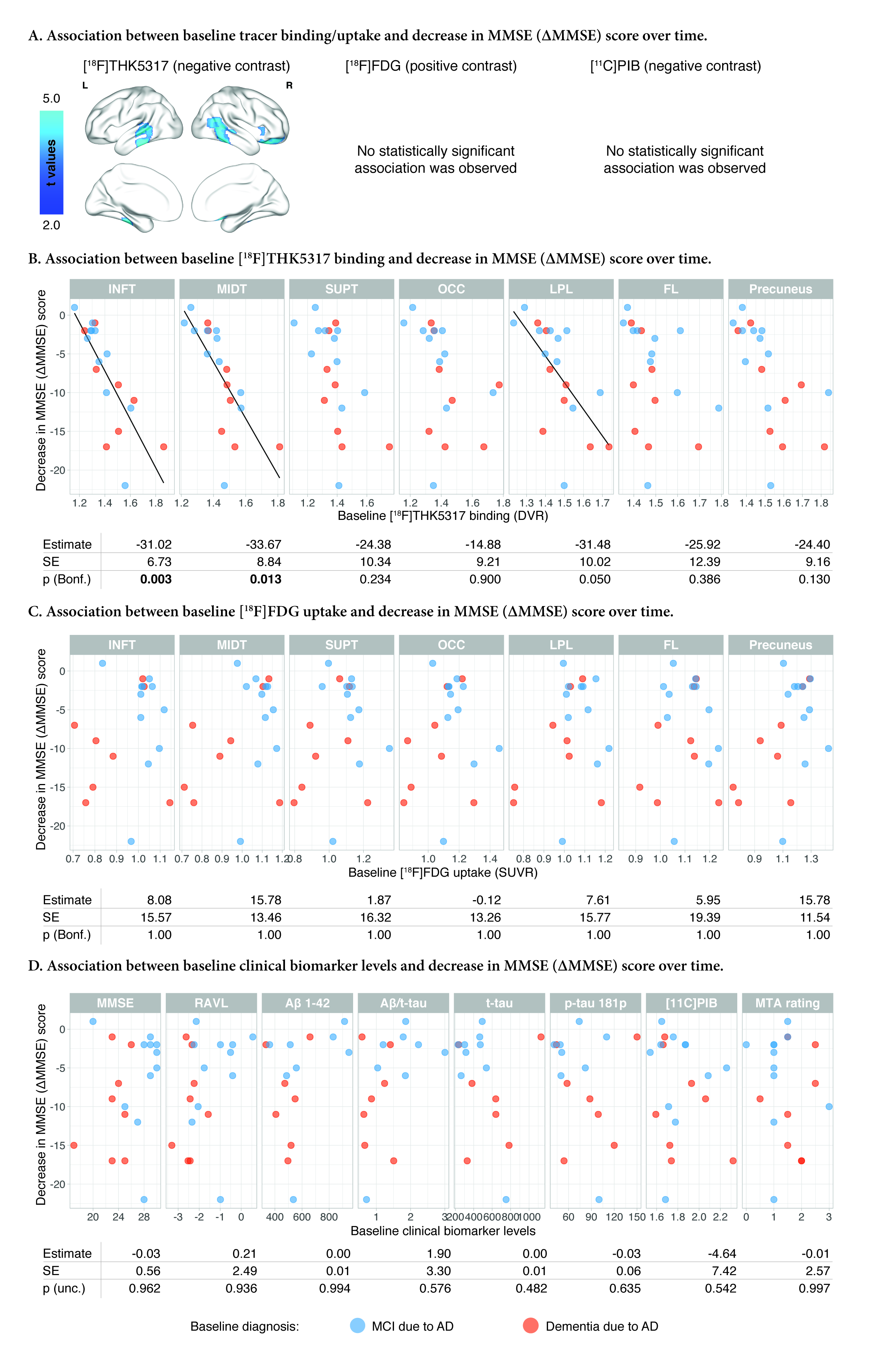


Supplementary Fig 2. Interaction plots of the relationship between baseline biomarker levels and MMSE scores at baseline and follow-up (biomarker level x time) as fitted by linear-mixed effects models after adjusting for baseline diagnosis and age. Differential relationships between baseline (A) regional [^18^F]THK5317 binding, (B) regional [^18^F]FDG binding, and (C) clinical biomarker levels and MMSE score at the different time points. The estimated interaction effect, the standard error (SE) and the p values are shown below the respective interaction plots. Bonferroni-corrected (Bonf.) p values based on the number of regions investigated (n=7) are presented for regional tracer binding/uptake results. Uncorrected (unc.) values are presented for the clinical biomarker results. FL, frontal lobe; INFT, inferior temporal gyrus; LPL, lateral parietal lobe; MIDT, middle temporal gyrus; OCC, occipital lobe; SUPT, superior temporal gyrus.


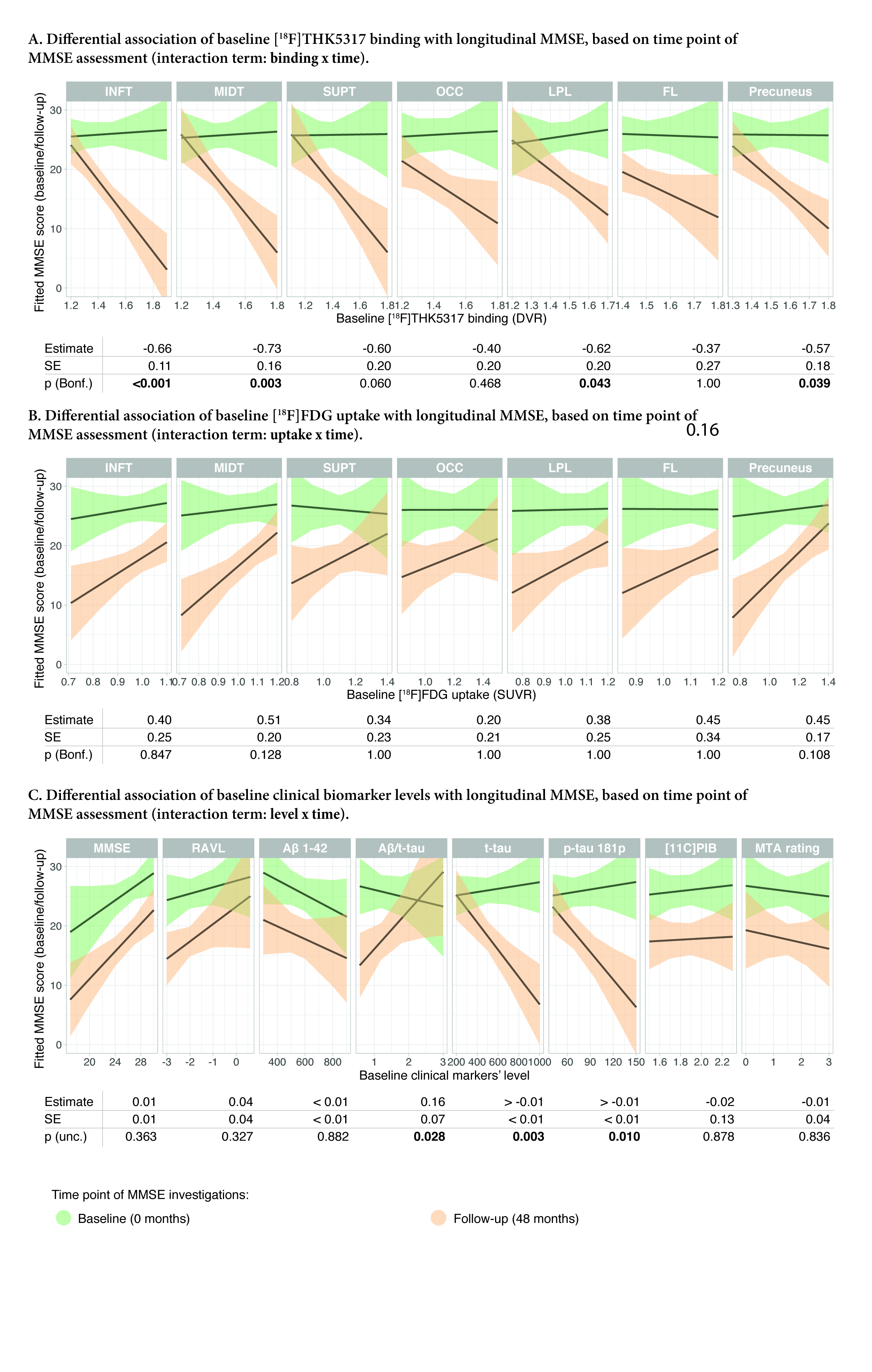


Supplementary Fig 3. Replication of regional analyses for [^18^F]THK5317 binding in the subgroup of patients with available CSF measures (n=16) for comparison purposes. (A) Dot and violin plots showing the levels of regional baseline [^18^F]THK5317 binding in patients who remained cognitively stable (STABLE) and those who declined cognitively (DECL.) over time. The calculated AUC and the sensitivity and specificity of the optimal cut-off points for classifying patients who remained cognitively stable or declined cognitively over time are shown below the respective dot and violin plots. (B) Scatterplots showing the relationships between regional baseline [^18^F]THK5317 binding and decreased MMSE scores after adjusting for baseline diagnosis and age. (C) Interaction plots of the differential relationship between baseline regional [^18^F]THK5317 binding and MMSE scores at baseline and follow-up (biomarker level x time) as fitted by linear-mixed effects models after adjusting for age. The estimated effect, the standard error (SE) and the p values are shown below the respective plots. Bonferroni-corrected (Bonf.) p values based on the number of regions investigated (n=7) are presented. FL, frontal lobe; INFT, inferior temporal gyrus; LPL, lateral parietal lobe; MIDT, middle temporal gyrus; OCC, occipital lobe; SUPT, superior temporal gyrus.


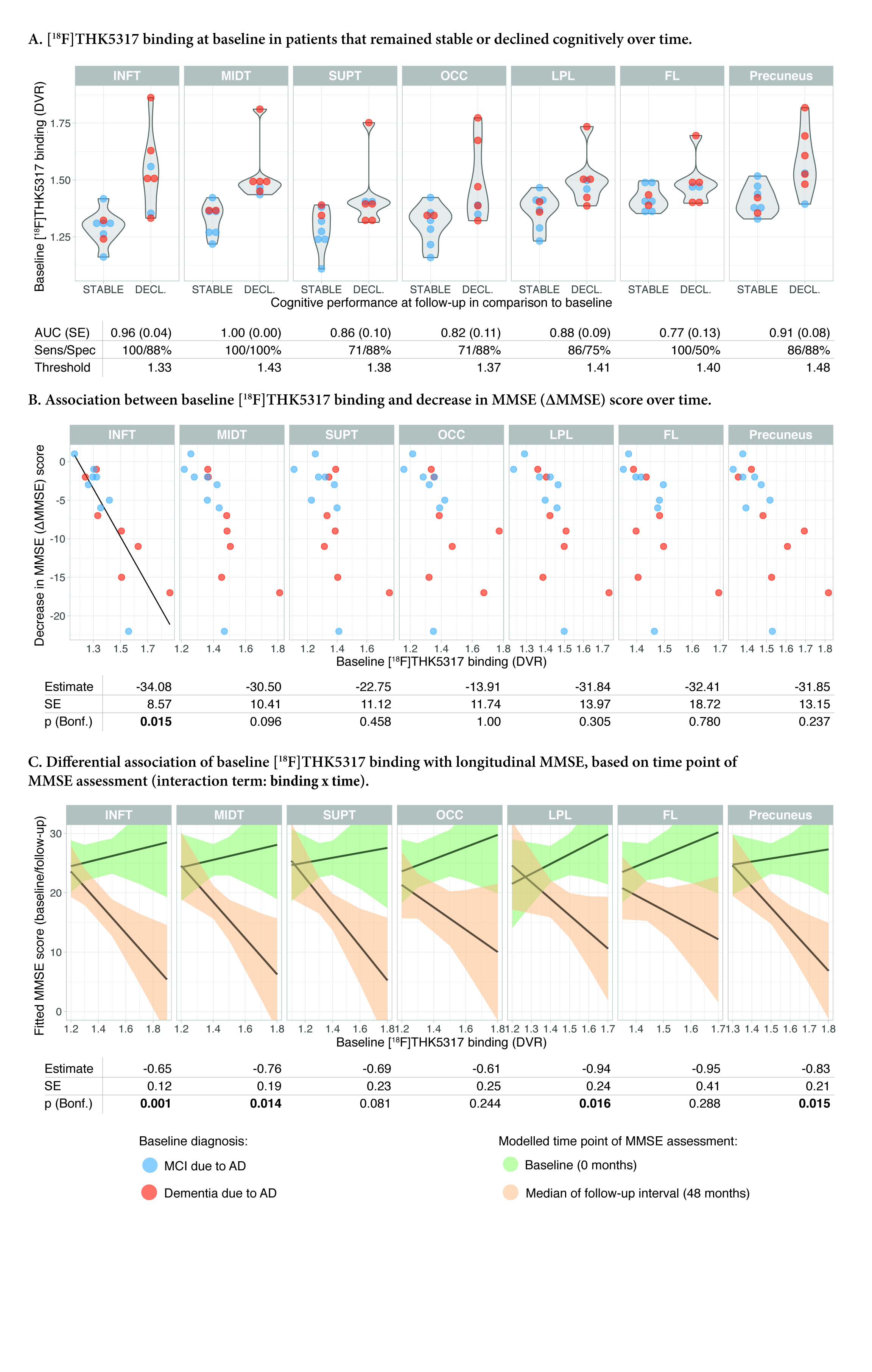

Supplement: Supplementary file 1 — Supplemental Material [file 41380_2020_815_MOESM1_ESM.docx]
